# Use of CBCT in dental implantology: a cross-sectional survey among postgraduate students in Italy

**DOI:** 10.1186/s12903-026-08206-9

**Published:** 2026-07-25

**Authors:** Antonio Scarano, Sergio Alexandre Gehrke, Gianna Dipalma, Ahmad G.A. Khater

**Affiliations:** 1 Department of Innovative Technologies in Medicine and Dentistry, University of Chieti-Pescara, Via Dei Vestini 31, Chieti, 66100 Italy; 2 Department of Biotechnology, Universidad Católica de Murcia (UCAM), Murcia, 30107 Spain; 3 Department of Interdisciplinary Medicine, University of Bari “Aldo Moro”, Bari, Puglia Italy; 4 Faculty of Dental Medicine and Oral Health Sciences, The Alan Edwards Centre for Research on Pain, McGill University, Montreal, QC Canada

**Keywords:** Dental Radiography, Cancer, Cone-Beam Computed Tomography, Patient Dose, Radiation Exposure, Diagnostic Imaging

## Abstract

**Aims and objectives:**

Cone-beam computed tomography (CBCT) is now widely used in dental practice as a 3D imaging modality, largely because it offers lower costs and reduced radiation exposure compared with conventional CT. However, this feature of accurate imaging with a lower radiation dose than CT led many clinicians to perform CBCT scans more frequently, even when there was no indication. The aim of this study was to investigate how postgraduate students in oral surgery and implantology use radiological imaging, including CBCT, during their diagnostic and operative workflows.

**Materials and methods:**

An online questionnaire was used to survey postgraduate students enrolled in the oral surgery and implantology programs at two Italian universities (Chieti and Bari) during the winter semester of 2024. Participation was entirely voluntary, with no rewards or penalties for participation. The questionnaire comprised various question formats, including yes/no, multiple choice, and close-ended questions. The questions were designed to cover various aspects of knowledge related to using CBCT in dental implants, along with the latest recommendations based on current evidence. Out of 650 invitations sent, 282 doctors fully completed the questionnaire, while the remaining 38 responses were excluded due to incomplete answers. We analyzed the associations between pairs of categorical variables using Pearson’s chi-square test of independence, in order to assess the statistical significance of the comparisons.

**Results:**

A highly significant association emerged between attendance at implantology training courses and the prescription of CBCT exams (*p* = 0.00066). Furthermore, professionals who report knowing the guidelines for prescribing radiological exams are statistically more likely to specify the field of view field of view and technical parameters (mA, kVp) when requesting the exam (*p* = 6.0452e-61). The use of virtual planning software for guided surgery is also significantly associated with participation in specific training courses (*p* = 0.0006623).

**Conclusions:**

Our findings revealed that, despite a high level of awareness of the presence of CBCT, practitioners often lacked an understanding of the negative consequences, indications, features, and radiation doses of CBCT that are associated with increased stochastic effects. This is mainly due to the misconception that CBCT is a low-dose test, which led many implantologists to prescribe needless CBCT and, even more concerning, follow-up radiographs.

**Supplementary Information:**

The online version contains supplementary material available at https://doi.org/10.1186/s12903-026-08206-9.

## Introduction

Following the discovery of X-rays in 1895, significant advancements were made in several fields of surgery; however, this also presented new challenges for surgeons and clinicians. Although the carcinogenic effect was recognized with large doses of X-rays, such as those used in fluoroscopy and radiotherapy, the effect of modest doses of X-rays used in diagnostic radiology was disregarded for many years [1, 2]. In dentistry, X-rays are primarily used for diagnosis by aiding in the detection of caries, impacted teeth, and bone disease, among other conditions, and for monitoring patients after treatments. Conventionally used X-ray modalities include intraoral (IO), panoramic (Pan), and cephalometric (Ceph) radiographs, which are two-dimensional images and have several limitations, such as superimpositions and distortions. In 1972, G.N. Hounsfield introduced Computed Tomography (CT) using transverse axial scanning; however, its use in dentistry has been limited due to its high cost and increased radiation exposure [3]. Afterward, Cone Beam Computed Tomography (CBCT) was introduced for oral and maxillofacial applications, as it provides three-dimensional (3D) imaging that is more accurate compared to 2D modalities [4, 5]. As a result, CBCT has become substantially involved in dental practices as 3D imaging due to its lower cost and decreased radiation doses, which are 6 to 15 times lower than those of 3D imaging using spiral CT [6, 7]. However, this feature of accurate imaging with a lower radiation dose than CT led many clinicians to perform CBCT scans more frequently, even when there was no indication. Although the adverse effects of X-rays, such as skin reddening and immediate damage, require high doses of X-rays (around 2 Gy), lower doses exposed during diagnostic investigations might also cause DNA damage and lead to the death of some cells [8, 9].

During dental implants planning, CBCT provides vital information regarding the morphological characteristics and orientation of the residual alveolar ridge, as well as the local anatomy and presence of lesions. CBCT may be recommended when the clinical situation requires it, after medical history, clinical examination, and 2D imaging, and by applying justification and optimization criteria [10]. Although these investigations are valuable and useful during practices, their application in patients must be justified after careful assessment, with precise clinical and surgical indications. Unfortunately, misuse of CBCT scans sometimes occurs in daily dental practices, exposing patients to excessive and unnecessary X-ray doses [11]. Notably, dental radio-diagnostics represent about 25% of all radiological exams performed in the EU; thus, these types of investigations necessitate particular consideration when it comes to radiation safety [12]. For instance, the individual exposure of patients to ionising radiation has doubled over the past 25 years. Historically, the distribution of dose exposure was 85% from natural exposure (e.g., cosmic rays, sun, and radon) and 15% from medical exposure (e.g., examinations or therapies with ionizing radiation) [13]. Today, individual exposure has substantially increased mainly due to the wider use of computed tomography (CT) and nuclear medicine, although trends in medical radiation exposure vary by region. In the United States, medical imaging has contributed to a substantial share of the population’s exposure (as detailed in NCRP Report No. 184) [14]. By contrast, global and European estimates are lower: UNSCEAR’s 2020/2021 evaluation reports a worldwide mean medical effective dose of ~ 0.57 mSv per caput (2009–2018), while the European Commission’s Radiation Protection Report No. 180 estimates ~ 1.1 mSv per caput across 36 European countries (2007–2010), with considerable inter‑country variability [15]. A 2022 systematic review and cross-sectional survey found that students, including postgraduate trainees, show variable levels of knowledge regarding the indications, radiation doses, and clinical applications of CBCT, with a general tendency toward limited awareness [16]. Recent literature highlights the increasing use of CBCT in implantology, describing its advantages and limitations while emphasizing the risk of overuse and unjustified prescriptions in straightforward cases. A 2024 clinical study confirmed the central role of CBCT in both pre- and postoperative phases; however, it also stressed the need for adequate informed consent and justified use to avoid unnecessary radiation exposure [17]. These findings are further supported by a 2025 study showing that many dentists have insufficient knowledge about CBCT and that examination prescriptions remain high despite limited formal training on how to perform and interpret CBCT imaging [18].

The aim of this study was to investigate how postgraduate students in oral surgery and implantology use radiological imaging, including CBCT, during their diagnostic and operative workflows.

## Materials & methods

### Study design

This study followed the STROBE guidelines for reporting cross-sectional observational studies [19]. An online questionnaire was used to survey postgraduate students in the oral surgery and implantology programs at the Italian universities during the winter semester of 2024. Participation was entirely voluntary, with no rewards or penalties for participation. Given that no personal data was collected, and participants’ anonymity was maintained, the Institutional Review Board at G. D’Annunzio University of Chieti-Pescara considered ethical approval unnecessary under local regulations and assessed the study as not requiring notification. Informed consent was not required under Italian Legislative Decree n. 196/2003 (Data Protection Code) and EU Regulation 2016/679 (GDPR), as participation was voluntary and the questionnaire was completed anonymously.

### Setting and participants

We employed convenience and snowball sampling methods by contacting the directors of postgraduate oral surgery and implantology programs to request a list of enrolled students. Based on student lists, a random sample was performed using computer-generated random numbers, using *Microsoft Excel’s RAND* function, based on student IDs to recruit full-time postgraduate students enrolled in the university’s oral surgery and implantology programs. The selected students were invited to participate via their institutional emails, with the emphasis that their participation is entirely voluntary for research purposes and has no impact on their studies or grades. We included full-time postgraduate students enrolled in clinical training programs who were actively practicing dental implantology and were expected to obtain a specialty license upon completing their program, allowing them to practice dental implants. Part-time students and those pursuing postgraduate research were excluded from the study.

### Data sources

A multidisciplinary team of experts (AS, G.D, A.K) in dental implantology and oral maxillofacial radiology, with over ten years of clinical and research experience, has developed a questionnaire to assess the use of CBCT among postgraduate students (See Supplementary File 1 for the questionnaire). The questionnaire comprises various question formats, including yes/no, multiple choice, and open-ended questions. The questions were designed to cover various aspects of knowledge related to using CBCT in dental implants, along with the latest recommendations based on current evidence. Additionally, the questions were designed to limit the responses so that they could be summarized and presented clearly. After drafting the initial questionnaire, its content was validated by assessing the intra-rater reliability of a pilot sample consisting of ten students from three different specialties (oral surgery, periodontics, and prosthodontics) with a one-month interval between the test and retest. We tested the correlation between the two rounds using the Pearson’s chi-square correlation and consequently removed questions with low reliability. The final version of the questionnaire was converted into a Google Form and distributed. The questionnaire began by outlining the study goals and emphasizing the voluntary nature of participation. The initial questions focused on demographic characteristics, followed by knowledge-based questions about the use of CBCT in dental implants.

### Sample size

To estimate the association between two categorical variables in a 2 × 2 contingency table, a sample size calculation was performed based on: Expected effect: medium (Cramer’s V = 0.3); Significance level (α): 0.05; Statistical power (1 - β): 0.80. Using the chi-square test for independence, the estimated sample size is 87 subjects. This value represents the minimum number necessary to detect a statistically significant association between the variables under consideration, with a risk of type I error of 5% and a power of 80%. A sample of 650 dentists attending or completing a postgraduate course at the university were randomly selected and sent a questionnaire.

### Statistical analysis

Descriptive statistics were used to present the participants’ responses as frequencies and percentages for categorical variables. To compare the study variables, Pearson’s chi-square correlation test was performed using the data. Differences were considered statistically significant if *p* < 0.05. All statistical analyses were performed using SPSS 28 (IBM., Armonk, USA).

## Results

### Participants’ demographic characteristics

Out of 650 invitations sent, 282 postgraduate students enrolled in oral surgery and implantology programs across two Italian universities (Chieti and Bari) voluntarily accepted to participate in the study. Of these participants, fully completed the questionnaire, while the remaining 38 responses were excluded due to incomplete answers.

Most of the participants were female, with a male-to-female ratio of 41.5% to 58.5%, and their ages ranged from 26 to 65 years old. Many of the participants were enrolled in the oral surgery programs (57.6%), and 50% of participants had more than five years of clinical experience.

A total of 282 postgraduate trainees and general dentists completed the survey (response rate: 43.4%; 282/650). Females accounted for 58.5% (*n* = 165) and males for 41.5% (*n* = 117). Overall, 37.8% (*n* = 107) identified as *postgraduates with institutional affiliation* and 62.2% (*n* = 175) as *general dentists with institutional affiliation*. Among postgraduates (denominator not directly available in the PDF summary), the reported specialties were Oral Surgery (57.6%), Periodontology (22.0%), and Prosthodontics (20.3%).

### Main findings

In the preoperative phase, most participants (74%) reported using intraoral radiographs for initial assessment, followed by panoramic radiographs (20.5%) and CBCT (5.5%).

**Fig. 1:**
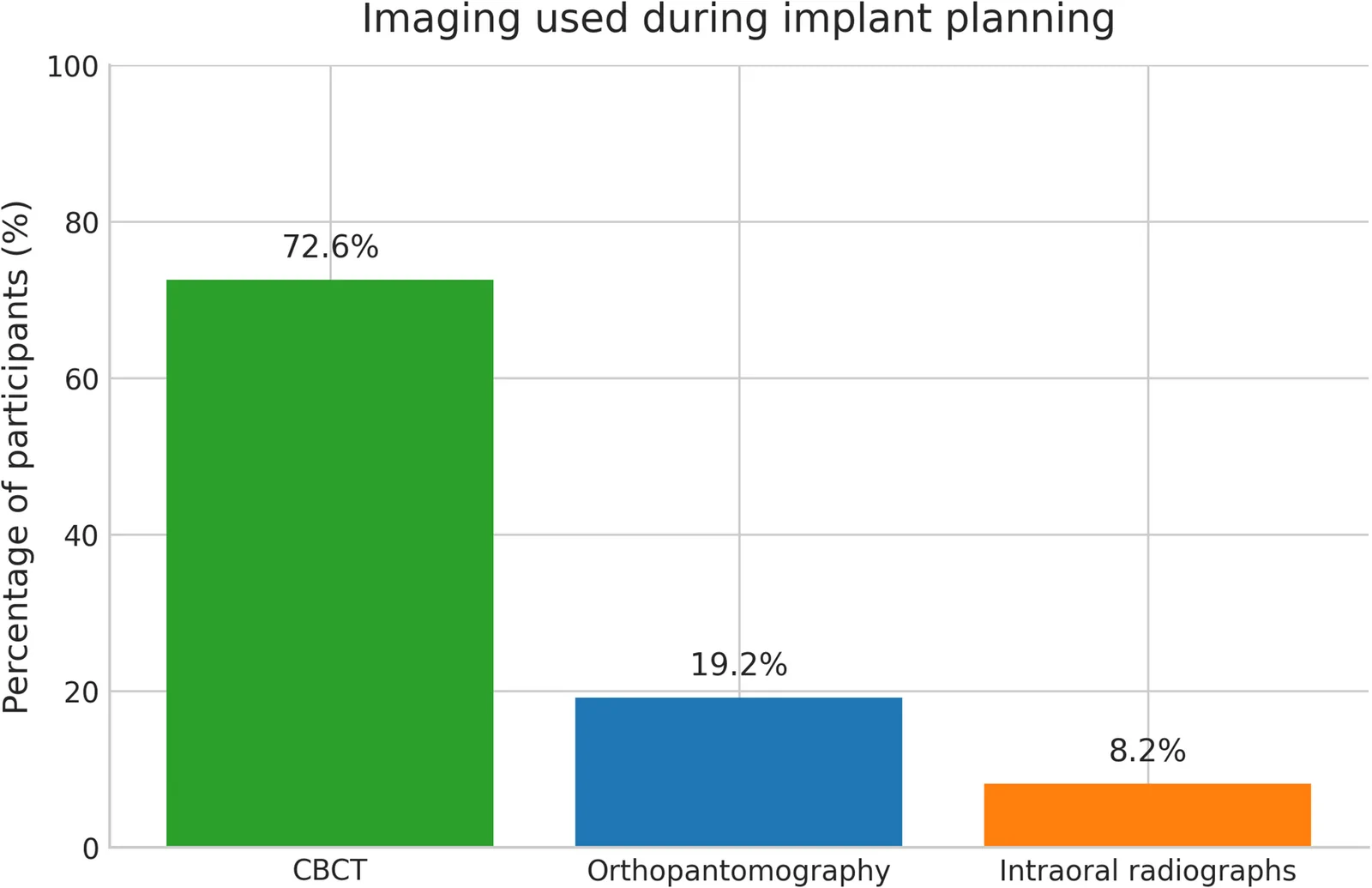
The x-ray chose doctors for evaluation and planning of implant placement

During the implant-planning phase, CBCT became the predominant modality (72.6%), whereas panoramic radiographs and intraoral radiographs were used less frequently (19.2% and 8.2%, respectively) (Fig. 1). A total of 282 postgraduate trainees and general dentists completed the survey (response rate: 43.4%; 282/650). Females accounted for 58.5% (*n* = 165) and males for 41.5% (*n* = 117). Overall, 37.8% (*n* = 107) identified as postgraduates and 62.2% (*n* = 175) as general dentists. Among postgraduates, the reported specialties were Oral Surgery (57.6%), Periodontology (22.0%), and Prosthodontics (20.3%).

**Fig. 2:**
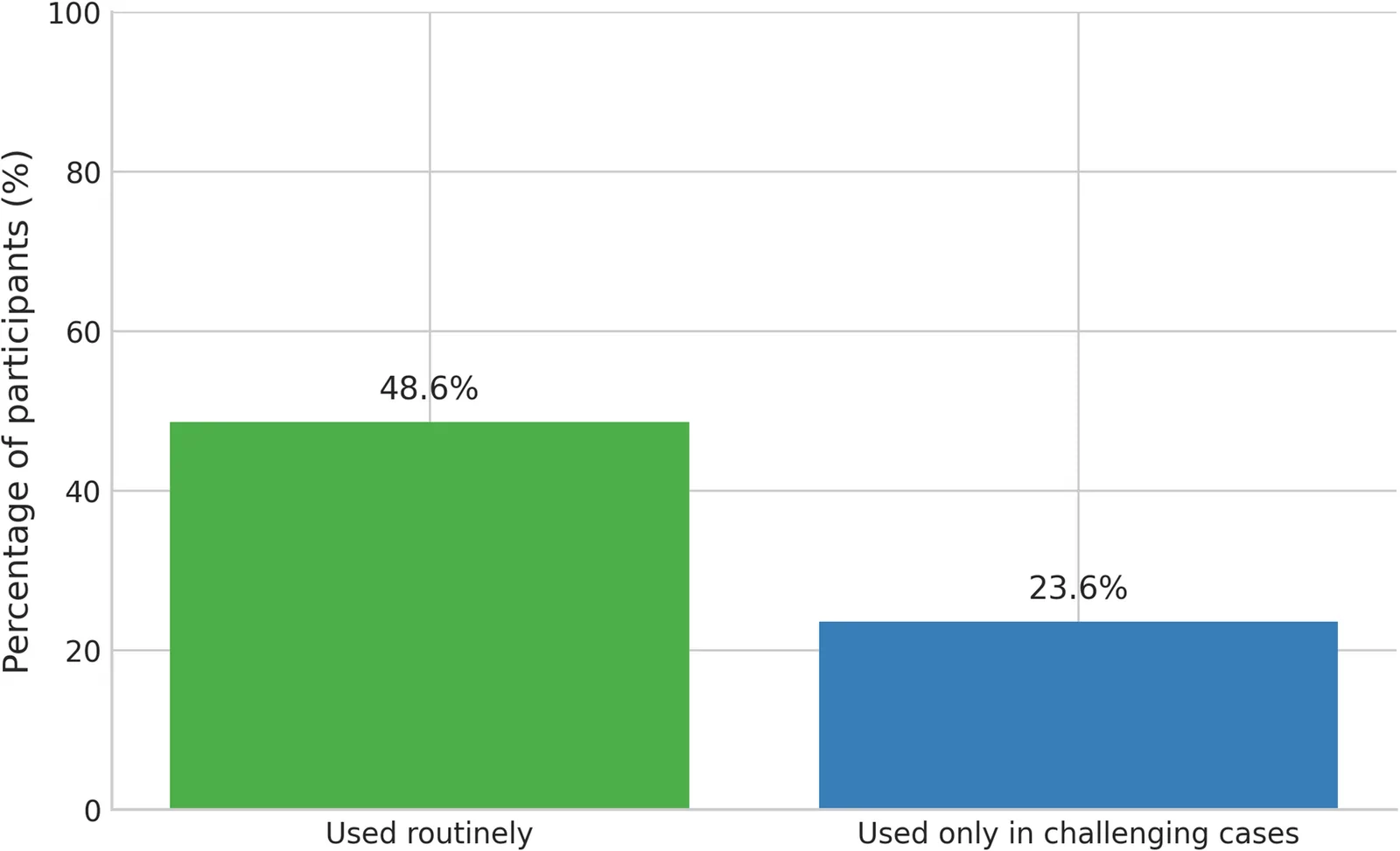
Use of Virtual Planning Software for Guided Implant Surgery. Used for all guided surgeries: 48.6%. Used only in challenging cases: 23.6%

**Fig. 3:**
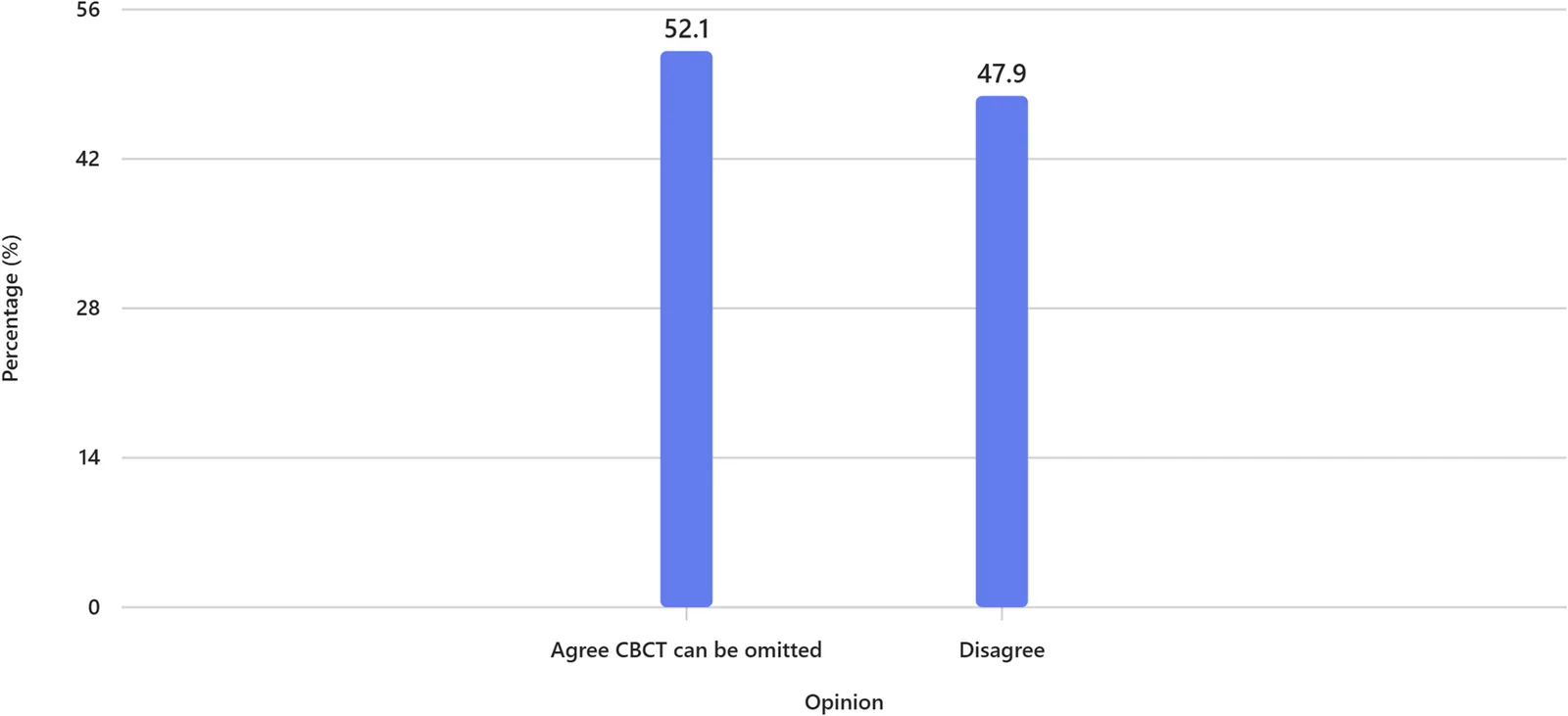
Opinion on omitting CBCT in straightforward clinical cases—defined as situations with sufficient bone thickness and height to allow implant placement without the need for any regenerative procedure. Agree CBCT can be omitted: 52.1%, Disagree: 47.9%

**Fig. 4:**
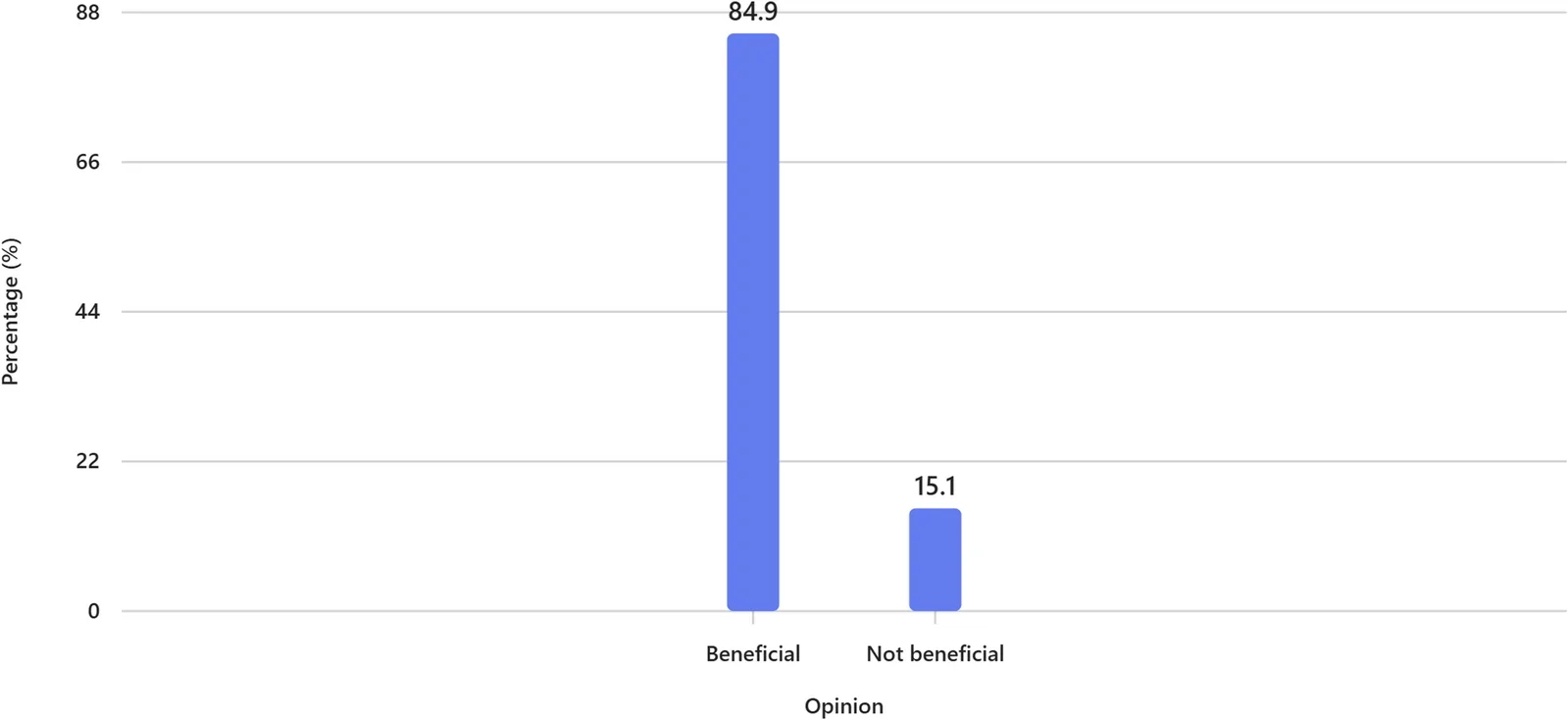
Radiographs Used in Immediate Postoperative Assessment. Beneficial: 84.9%. Not beneficial: 15.1%

**Fig. 5:**
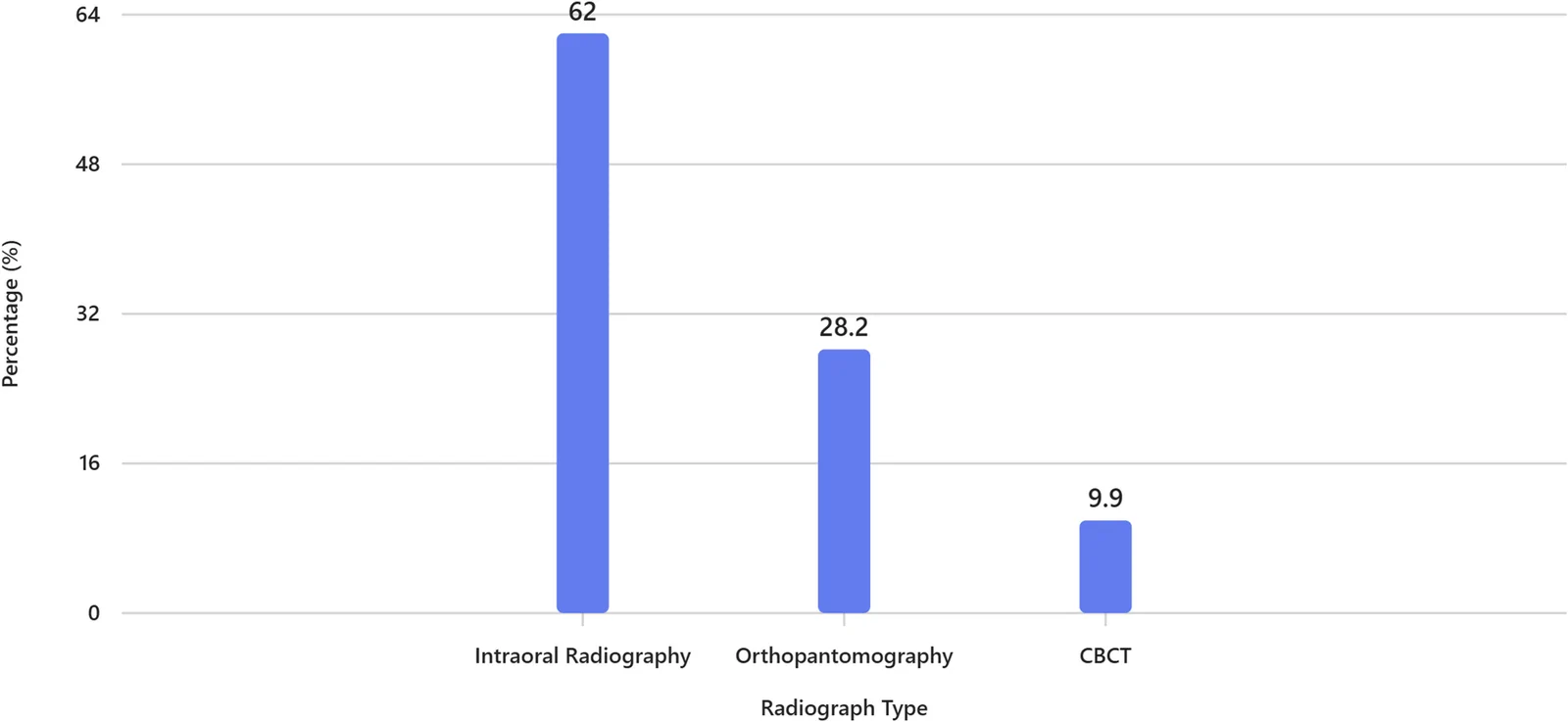
Radiographs chose for follow-up the most used radiographs in postoperative assessment during follow-up

Additionally, 48.6% of participants employed virtual planning software for guided implant surgery, while 23.6% used it only in challenging cases (Fig. 2). Notably, 52.1% of participants believed that CBCT could be omitted in straightforward clinical cases, while 47.9% disagreed (Fig. 3). On average, patients underwent five radiographs before treatment. In the immediate post operative phase, most participants (84.9%) believed that postoperative radiographs are beneficial, while 15.1% disagreed (Fig. 4). In follow-up, the most used radiographs in postoperative assessment were intraoral radiography (62%), orthopantomography (28.2%), and CBCT (9.9%) (Fig. 5 and Table 1). We analyzed the associations between pairs of categorical variables using Pearson’s chi-square test of independence, in order to assess the statistical significance of the comparisons. A highly significant association emerged between attendance at implantology training courses and the prescription of CBCT exams (*p* = < 0.001). Furthermore, professionals who report knowing the guidelines for prescribing radiological exams are statistically more likely to specify the field of view (FOV) and technical parameters (mA, kVp) when requesting the exam (*p* < 0.001). The use of virtual planning software is also significantly associated with participation in specific training courses (*p* = < 0.001). Gli specialisti utilizzano la CBCT per il planning implantare molto più spesso dei non‑specialisti, in modo statisticamente significativo (*p* < 0.001).

**Table 1:** Survey Results, 282 responses to the 20 administered questions

Category	*n*	%
Question 1 – Gender Distribution		
Male	165	58.5%
Female	117	41.5%
Question 2 – Implant Training		
Yes	186	65.9%
No	96	34.1%
Question 3 – Qualification		
Postgraduate professional	175	62.2%
General dentist	107	37.8%
Question 4 – Postgraduate Specialization		
Oral Surgery	101	57.6%
Periodontology	39	22.0%
Dental Prosthesis	35	20.3%
Question 5 – Radiographic Exam (General Assessment)		
Orthopantomography	209	74.0%
Intraoral radiography	58	20.5%
CBCT	15	5.5%
Question 6 – Radiographic Exam (Implant Planning)		
CBCT	205	72.6%
Orthopantomography	54	19.2%
Intraoral radiography	23	8.2%
Question 7 – CBCT Omission in Simple Cases		
Yes	147	52.1%
No	135	47.9%
Question 8 – Postoperative Radiographic Examination		
Yes	239	84.9%
No	43	15.1%
Question 9 – Virtual Planning Software Use		
Yes	137	48.6%
No	78	27.8%
Only difficult cases	67	23.6%
Question 10 – Implant Method Awareness		
Submerged	160	56.9%
Immediate loading	82	29.2%
One-stage	39	13.9%
Question 11 – CBCT Prescription Guidelines		
Yes	219	77.8%
No	63	22.2%
Question 12 – Specify FOV/Exposure Parameters		
Yes	145	51.4%
No	137	48.6%
Question 13 – Familiar with Virtual Planning Software		
Yes	185	65.7%
No	97	34.3%
Question 14 – Awareness of Implant Body Configurations		
Yes	231	81.9%
No	51	18.1%
Question 15 – Implant Placement Approach		
Two-stage	164	58.3%
One-stage	118	41.7%
Question 16 – Age Group of Implant Patients		
Middle-aged	208	73.6%
Elderly	59	20.8%
Younger	15	5.6%
Question 17– Feel Competent to Place Implants		
Yes	227	80.6%
No	55	19.4%
Question 18 – Need Additional Training		
Yes	227	80.6%
No	55	19.4%
Question 19 Awareness of Implant Surface Modifications		
Yes	242	85.9%
No	40	14.1%
Question 20 Summary of Radiographic Frequency (Selected Key Data)		
Intraoral radiographs annually (1)	93	32.9%
Orthopantomographs annually (1)	147	52.1%
CBCT annually (1)	93	32.9%

## Discussion

The results of the questionnaire administered to 282 dentists show that the CBCT exam is widely prescribed, particularly by those who have attended a training course in implantology, by those who perform one stage implant surgery, and especially by those who use the computer-guided technique. CBCT has already proven to be a reliable diagnostic tool for a wide range of dental indications, including orthodontics, endodontics (both surgical and non-surgical treatments), preoperative planning of periodontal surgery, forensic odontology, dental traumatology, evaluation of periodontal bone defects, evaluation of paranasal sinuses, bone quality assessment, and dental implant planning. The present survey highlighted an important initial finding regarding the progressive shift from conventional 2D imaging to CBCT throughout the diagnostic workflow. Although intraoral radiographs remain the preferred option during the initial assessment (74%), their use decreases considerably in the planning phase, where CBCT becomes the predominant modality (72.6%), acknowledging its ability to provide accurate information on bone morphology, anatomical structures, and implant positioning. This trend is consistent with European and international guideline recommendations, which support CBCT use when three‑dimensional information is essential but discourage routine application in simple cases. The limited use of CBCT (5.5%) during the first visit suggests that clinicians generally reserve it for more advanced planning stages, in accordance with the principles of justification and dose optimization.

Our study also highlights a significant increase in the use of digital tools: 48.6% of participants routinely use virtual planning software for implant surgery, while an additional 23.6% employ it selectively in complex cases. This trend underscores the growing confidence in computer‑assisted workflows, which have been shown to improve accuracy and safety in implant placement.

Notably, opinions on the need for CBCT in simple clinical cases are nearly evenly divided: 52.1% believe it can be omitted, whereas 47.9% disagree. This polarization reflects persistent uncertainty within the profession, likely linked to differences in training, risk perception, and knowledge of radiological guidelines. Our study also showed that patients underwent an average of five radiographic examinations throughout the diagnostic‑therapeutic pathway, further emphasizing the importance of promoting a culture of dose optimization.

Postoperative and follow‑up practices also show considerable heterogeneity. The vast majority of clinicians (84.9%) consider postoperative imaging useful, most frequently relying on intraoral radiographs (62%) during follow‑up, while panoramic radiography (28.2%) and CBCT (9.9%) are used less often. This distribution suggests that CBCT is generally reserved for specific postoperative situations, such as complications, whereas 2D radiography remains the standard for routine monitoring.

Pearson’s chi‑square analysis showed an association between participation in implantology training courses and a greater prescription of CBCT examinations. This indicates that postgraduate training may increase clinicians’ awareness of the usefulness of 3D imaging—probably mainly in relation to surgical safety and possibly also for medico‑legal reasons. The key elements determining the choice of imaging protocol are the clinical situation’s complexity and each practitioner’s preferred practice pattern, such as virtual planning software for guided implant surgery. However, guideline recommendations should also be considered in clinical decision-making. For instance, CBCT was indicated for patients with more than eight to ten sites, although there is currently no high-certainty evidence to support this [20]. Therefore, it will be challenging to establish that CBCT is statistically and demonstrably superior to traditional two-dimensional imaging modalities, such as panoramic radiography, in terms of preventing injury to the IAN or other important neurovascular structures [10, 21]. As a result, when prescribing CBCT, implantologists should identify and consider the imaging aim for the patient’s particular situation, including exposure (mA and kVp), minimum image quality criteria, and FOV restriction to appropriately visualize the region of interest [22]. Implantologists should weigh the pros and cons of various imaging modalities and personalize radiographic examinations to each patient’s individual diagnostic needs. The mA and kVp parameters depend on the technological characteristics of each CBCT device, and therefore their specification by the clinician has relative importance. Conversely, defining the field of view (FOV) retains crucial clinical relevance, as it remains an essential parameter for radiation dose optimization.

Although diagnostic aims differ based on the cases, the following main themes should be used to prescribe radiological images: 1) Imaging can assist in answering diagnostic questions. 2) Minimize radiation exposure for patients while obtaining diagnostic information. 3) The diagnostic investigation’s benefits should outweigh the projected radiation risks. As such, applying these principles helps clinicians make decisions and assures the safety and utility of radiographic imaging [23–25].

In this regard, dentists might be accountable for the rising incidence of cases with head and neck cancers due to excessive intraoral radiographs and CBCT [26]. The International Commission on Radiological Protection has issued guidelines on CBCT, suggesting sensible CBCT usage in dental practices and favouring other imaging modalities [27]. Notably, dentists prescribe more than 46% of imaging and nuclear medicine procedures performed nationwide, which include panoramic, intraoral, and CBCT radiographs [28]. Each sort of ionizing radiation can raise the chance of damaging health consequences, which are typically divided into two categories: deterministic impacts and stochastic (random) effects [29]. The natural background radiation to which humans are exposed is approximately 6–10 microsieverts (µSv) per day. However, the dosage absorbed by performing an intra-oral radiograph is 5–10 µSv, while an orthopantomography yields 4–30 µSv, and a CT scan ranges from 100 to over 2000 µSv [30, 31]. Radiation exposure of ≥ 100 mGy can cause tissue responses, skin burns, cataracts, or cellular death. These consequences are not seen in diagnostic medicine. Exposure levels from dental imaging (low-level radiations) are often much below this threshold; yet, they have stochastic consequences. These stochastic consequences, such as heritable or genetic alterations and cancer, are always there regardless of the initial dosage. This stochastic impact occurs independently of the dose and is in addition to the harm caused by prior radiographs, resulting from the numerous X-ray investigations that patients undergo throughout their lifetimes. Low-level radiation exposure has been associated with the development of oxidative stress, potential DNA damage, and an increased risk of carcinogenesis [32].

The radiation causing stochastic effects leads to variations and damage in DNA sequences. Such DNA alterations in somatic cells can result in neoplasia, and the risk of radiation-induced neoplasia in individuals exposed to ionizing radiation is well-documented [33–35]. Therefore, CBCT should be restricted in complicated cases and never be used routinely in oral surgeries when alternative lower-dose radiography modalities could provide sufficient diagnostic data [36, 37], as frequently using CBCT for dental diagnostics may increase the risk of causing genetic damage. Recent research using micronuclei formation revealed that CBCT radiographs during dental examinations had cytotoxic and genotoxic effects on exfoliate buccal epithelial cells, which increased with increased field of view FOV [38, 39]. Following CBCT, chromatin material particles formed micronuclei in buccal cells and peripheral lymphocytes [40, 41]. As a result, damaged or defective spindle chromosomes cannot travel to the poles during anaphase and are not integrated into the nucleus of a dividing cell during telophase [42]. Thus, an FOV increase from 6 × 6 to 6 × 11 cm2 could cause an increase in cytotoxicity and genotoxicity in the oral mucosa without regard to gender. Additionally, the peak voltage supplied to the X-ray tube (kVp), electron flow through the X-ray tube (mA), and voxel size [43] all have an impact on the dosage received by patients. This is why it is critical to use the ALARA (as low as reasonably achievable) [43] and ALADA (as low as diagnostically acceptable) [44] principles when utilizing CBCT to diagnose dental diseases. Thus, European and US guidelines recommend using CBCT only when truly necessary and justified on an individual basis, preferring CBCT over CT when cross-sectional imaging is required, while limiting the FOV and optimizing exposure parameters [45]. Therefore, the oral surgeons must optimize the exposure settings on an individual basis, maximizing the ability of their equipment to provide diagnostically valid images while exposing the patient to the lowest feasible radiation dosage.

Previously, thyroid shielding was recommended for individuals undergoing CBCT scans, especially when the FOV is large. However, the American Dental Association (ADA) recently advocated against employing a protective collar to shield the thyroid gland during intraoral radiography on adults or children [46]. Thus, thyroid collars are no longer indicated for intra-oral, panoramic, cephalometric, and CBCT radiographs because they do not protect against internal scatter radiation and induce artifacts, necessitating a repeat diagnostic examination [47]. Likewise, a European consensus on patient contact shielding recommended against using a collar during routine X-rays such as panoramic, intraoral, cephalometric, and CBCT imaging [48]. Additionally, the American Thyroid Association (ATA) considered protecting the thyroid gland with a collar during a dentomaxillofacial X-ray unnecessary [49]. As a result, the most effective approaches to reducing radiation doses to patients are to use rectangular collimation appropriately, position patients optimally for imaging procedures, and employ suitable dose-reduction strategies [47]. It has been established that focusing the x-ray beam to a specific area of interest, particularly when using rectangular collimation, may consistently reduce dosage by more than 40% [50, 51]. Another approach to reduce X-ray exposures is to examine a small region (e.g., 5/6 teeth, a full arch, or half of the mouth), which exposes oneself to doses ranging from 5 to 600 microsieverts, but a wide-area CT scan of the entire head can increase the dosage to 1400 microsieverts. Such excessive and incorrect exposures may raise the risk of thyroid diseases or some forms of cancer.

As a result, the guidelines recommended that dentists use X-rays only when truly necessary. According to the international guidelines, children should have no more than one intra-oral X-ray per 1–2 years, adolescents every 1.5-3 years, and adults every 2–3 years [52–54]. Additionally, clinicians should only perform radiographic imaging, such as CBCT, if they have reached the professional judgment that the imaging exam provides a clear clinical benefit that outweighs the risks associated with ionizing radiation exposure [28, 52]. In 2018, the Italian Society of Periodontology and Implantology (SIDP) released a statement on the needless prescription of CBCT in implantology, criticizing careless and incompetent use of the equipment. For instance, the four mini-intraoral X-rays are an excellent basic screening investigation that any dental practice can provide and involve very low radiation exposure: the natural background radiation to which we are exposed is eight µSv per day, and the additional dose from four intraoral X-rays is only five µSv [55]. Even orthopanoramic X-rays are low-dose, exposing humans to 3–24 microsieverts; in contrast, CT scans may vary from 280 to 1400 microsieverts, with the newer volumetric cone beam from 60 to 1000 [55]. According to the Italian Society of Medical Radiology (SIRM), out of over 40 million radiological exams performed annually in Italy, approximately 44% are incorrectly recommended and not necessarily essential [56]. Our results are consistent with numerous studies demonstrating variable knowledge levels and inconsistent prescribing behaviors regarding CBCT among dental professionals. A cross-sectional survey and a systematic review focusing on dental students, including postgraduate cohorts, reported heterogeneous understanding of CBCT indications, radiation doses, and clinical applications, with a general tendency toward limited awareness [18]. These findings support our observation that knowledge of guideline-compliant parameters is not uniform across practitioners. Similarly, a recent study found that many dentists show insufficient knowledge while frequently prescribing CBCT, highlighting a knowledge-practice gap that aligns with our results [16–18]. However, it is important to emphasize that this study highlights the need to also consider radiation effects when prescribing CBCT scans, pending further evidence on the advantages and disadvantages of the inevitable increase in CBCT prescriptions associated with computer-assisted implant planning.

## Conclusion

Our findings revealed that, despite a high level of awareness of the presence of CBCT, practitioners often lacked an understanding of the negative consequences, indications, features, and radiation doses of CBCT that are associated with increased stochastic effects. This is mainly due to the misconception that CBCT is a low-dose test, which led many implantologists to prescribe needless CBCT and, even more concerning, follow-up radiographs. Additionally, using software-assisted implant surgery encourages implantologists to prescribe CBCT scans when they already have adequate current panoramic images to complete the procedure. Furthermore, it should be noted that, to date, the digital workflow offers numerous benefits, the most significant being enhanced safety in avoiding errors and, of course, the correct positioning of implants.

## Supplementary Information

Supplementary Material 1.

## Data Availability

The datasets used and/or analysed during the current study are available from the corresponding author on reasonable request.
