# Supplementary material for: Use of CBCT in dental implantology: a cross-sectional survey among postgraduate students in Italy
Source: BMC Oral Health. 2026 Jul 25;26:2188. doi: 10.1186/s12903-026-08206-9 (PMC13732951; doi:10.1186/s12903-026-08206-9)
Supplement: Supplementary file 1 [file 12903_2026_8206_MOESM1_ESM.docx]

This is an anonymous survey; precious information will be kept confidential Please tick in the boxes provided.

- Gender: Male	Female
- Age:	years.
- Years of experience in the profession:	years.
- Implant training received:	Yes	No
- Post Graduate	Institutional based Practitioner

Institutional based Non‑Practitioner	General Dental Practitioner

- For PostGraduate: Specialization:

Oral Surgery	Periodontics	Prosthodontics

**QUESTIONNAIRE**

**KNOWLEDGE BASED QUESTIONS:**

1. Which radiographic investigation you use as a general evaluation?
   a) endoral X-ray	b) orthopantomography c) CBCT
2. Which radiographic investigation you use as a dental implant planning?
   a) endoral X-ray	b) orthopantomography	c) CBCT
3. Do you think that CBCT can be omitted in simple clinical cases?
   a) Yes	b) NO
4. Do you find it useful to perform a postoperative X-ray examination?
   a) Yes	b) NO
5. If so, which examinations do you use.
   a) endoral X-ray	b) orthopantomography	c) CBCT
6. Routinely uses virtual planning software for guided implant surgery.
   a) Yes	b) NO	c) Only for difficult cases (	%)
7. Which implantation method are you aware of ?
   a) emerged	b) submerged	c) immediate loading.
8. How many endoral X-rays do your patients take and how often per year.
   a) 0 b) 1	c) 2	3 d) one every 2 years	e) one every 3 years f) one every 3 years.
9. How many orthopantomography do your patients perform and how often per year.
   a) 0	b) 1	c) 2	3 d) one every 2 years	e) one every 3 years	f) one every 3 years.
10. How many CBCT do your patients perform and how often per year.
    a) 1	b) 2	c)	3 d) one every 2 years	e) one every two years	f) one every 2 years.
11. How many x-rays your patient took before your treatment.
    a) N°……endoral X-ray	b) N°……orthopantomography	c) N°….CBCT
12. There are guidelines on the prescription of CBCT
    a) Yes	b) No
13. When prescribing CBCT you also specify the FOV and exposure parameters (mA and kVp).
    a) Yes	b) No
14. Are you aware of various virtual planning software for guided implant surgery?
    a) Yes	b) NO
15. Are you aware of various body designs of implants?
    a) Yes	b) No
16. Which approach do you use for implant placement?

a )One stage	b) Two stage

17. The patients in whom you place implants lie in which age group mostly?

a) Younger	b) Middle aged

c) Elderly

18. Do you feel you are competent enough to place implants?
    a) Yes	b) No
19. Do you feel need for attending any training courses?
    a) Yes	b) No
20. Are you aware of various surface modifications of implants?
    a) Yes	b) No	.

*Thank you*
